# What we know so far and what we can expect next: A molecular investigation of plant parasitism

**DOI:** 10.1590/1678-4685-GMB-2024-0051

**Published:** 2024-09-30

**Authors:** Juliane Karine Ishida, Elaine Cotrim Costa

**Affiliations:** ¹ Universidade Federal de Minas Gerias (UFMG), Instituto de Ciências Biológicas, Departamento de Botânica, Belo Horizonte, MG, Brazil.; 2 Universidade Federal do Rio Grande (FURG), Instituto de Ciências Biológicas, Rio Grande do Sul, RS, Brazil.

**Keywords:** Parasitic plant, HGT, haustorium, plastid genome, mitochondrial genome

## Abstract

The review explores parasitic plants’ evolutionary success and adaptability, highlighting their widespread occurrence and emphasizing the role of an invasive organ called haustorium in nutrient acquisition from hosts. It discusses the genetic and physiological adaptations that facilitate parasitism, including horizontal gene transfer, and the impact of environmental factors like climate change on these relationships. It addresses the need for further research into parasitic plants’ genomes and interactions with their hosts to better predict environmental changes’ impacts.

## Introduction

Windsor (1998) asserts that the parasitic lifestyle is the prevailing survival tactic on Earth. Notably, even among flowering plants, this strategy is found, with approximately 292 genera and 4750 species capable of exhibiting parasitic behavior (Nickrent, 2020). The repeated occurrence of parasitism in plants, seen around 12 or 13 times throughout evolution (Westwood et al., 2010), emphasizes its remarkable adaptative capability and versatility. Indeed, this group is present in many ecosystems, including lush tropical forests and challenging habitats like dry and semi-arid areas, plains, savannas, and wetlands (Press and Graves, 1995). Their ability to adapt and use different survival tactics demonstrates their outstanding potential to navigate and thrive in response to various ecological stresses efficiently. 

A common feature shared by all plants that adopted this alternative nutrient-acquisition strategy is the presence of a new organ known as haustorium (plural haustoria). This organ is the primary interface for exchanging materials between the parasite and its host plant. The structure, typically a modified root or stem, forms a physical and physiological link that aids the attachment of parasitic plants to their hosts, enabling them to draw water, nutrients, and other vital resources (Yoshida *et al.,* 2016). Haustoria are the defining feature of parasitic plants. For instance, the mycoheterotrophs that do not form haustoria are not classified as true parasitic plants (Hershey, 1999). Mycohererotrophs are plants adapted to survive in dimly lit forests by deriving nutrients through parasitism on mycorrhizal fungi (Merckx et al., 2009). Parasitized fungi have a symbiotic relationship with photosynthetic plants. In this parasitism, mycoheterotrophs indirectly get organic carbon from green plants, using a fungus as an intermediary. Parasites exhibit higher transpiration rates (Ackroyd and Graves, 1997; Bell et al., 2011; Amutenya et al., 2023). It increases suction tension between the host-parasite link, creating a gradient that aids water transport by dragging nutrients towards the parasite in the xylem sap (Ackroyd and Graves, 1997; Jiang et al., 2003; Jiang *et al.,* 2007). *Rhinanthus minor* and *R. alectorolophus* (Orobanchaceae) maintain open stomata throughout the day and night as a transpirational sink to maximize xylem sap extraction from its host plants (Jiang *et al.,* 2007; Světlíková et al., 2018). 

The specific structure and function of the haustorium vary depending on the type of parasitism. The haustorium in holoparasitic plants is the primary means for acquiring all necessary nutrients from the host through phloem-based connections, compensating for their inability to photosynthesize autonomously due to the lack of chlorophyll (Nickrent and Musselman, 2004). Conversely, in hemiparasitic plants, the haustorium aids in absorbing water and nutrients from the host (Nickrent and Musselman, 2004). Although this parasite can engage in photosynthesis, the rates often remain limited in some species compared to nonparasitic plants. Hemiparasites can be further categorized into obligate and facultative parasites based on the degree of dependency on the host. An obligatory parasitic plant cannot complete its life cycle without establishing a connection with a host. On the other hand, a facultative parasitic plant can live as an autotrophic plant, but it will benefit from having a nearby host. Another distinguishing feature among parasites is the extent of parasitic tissues that develop inside and externally within the host. Most species introduce specialized cells into their host to provide nourishment for the parasite. However, in certain species, vegetative growth primarily occurs within the host organism (endophytically), with the parasite emerging just for the purpose of flowering (Thorogood et al., 2021).

Scientific investigations have advanced in the last 15 years and provided a deeper understanding of the complex molecular and physiological adaptations that enable parasitic plants to thrive. Studying how parasitic plants establish and maintain their reliance on host plants offers valuable insights into the intricate relationships and interdependencies within natural ecosystems. This review provides an overview of the current understanding of the modifications occurring in these plants’ nuclear, mitochondrial, and plastid genomes, which have enabled them to achieve repeated success throughout evolution. We examine the current understanding of the haustorium induction mechanism and the extrinsic elements that can impact its development.

## How did parasitic plants evolve?

The growing amount of large-scale sequencing information on parasitic plants enables us to address essential inquiries about the modifications and evolutionary mechanisms that enabled the shift from nonparasitic to parasitic behavior. At least 12 independent evolutionary occurrences of plant parasitism have been documented (Westwood et al., 2010), leading to the question of how parasitic plants managed to evolve multiple times during angiosperm evolution. This observation has sparked the hypothesis that common strategies might underlie the independent evolution of parasitic plants. In support of this argument, the genomes of distantly related lineages of parasitic plants (*Striga asiatica*, *Scurrula parasitica*, *Cuscuta australis*, two species of *Balanophora*, and *Sapria himalayana*) have shared genetic alterations that indicate a tendency towards parasitism (Chen et al., 2023). Some species exhibiting high levels of host dependency often experience a progressive loss of genes associated with carbon synthesis, circadian rhythm, flower and root development, nitrogen transporters, and Abscisic acid (ABA) biosynthesis (Chen *et al.,* 2023). The lack/reduced ability to synthesize ABA and respond to the circadian rhythm is intriguing. The genome comparison of mycoheterotrophic orchids *Gastrodia elata* and *Apostasia shenzhenica* (Zhang et al., 2017) with parasitic plants (*C. australis, S. asiatica,* and *Sapria himalayana)* revealed a reduction in homologs associated with functional categories such as photosynthesis, light perception, circadian clock, flowering time regulation, nutrient uptake, leaf, and root development (Xu *et al.,* 2021). Parasitism syndrome appears to promote a decreased sensitivity to environmental changes. As the parasite establishes a closer relationship with its host, it becomes increasingly dependent on the host’s external perception. 

Another significant aspect that contributes to the success of parasitism is the frequency of horizontal gene transfer (HGT) (Ding *et al.,* 2012). The HGT is a prevalent mechanism for directly introducing new genes to recipient species that are distantly related (Wickell and Li, 2020; Ma et al., 2022). As a result, this process can lead to the emergence of novel features, which in turn can contribute to the adaptation of species to new ecological niches (Ding *et al.,* 2012; Wickell and Li, 2020; Ma *et al.,* 2022). HGT has been demonstrated in the nuclear genomes of parasitic plants (Yoshida et al., 2010), as well as in their mitochondrial (Mower et al., 2004; Mower *et al.,* 2010; Xi et al., 2013) and plastidial (Sanchez-Puerta et al., 2023) genomes. The nuclear genome of eukaryotic cells differs from organellar genomes in mitochondria and plastids. Mitochondrial DNA (mtDNA) and plastid DNA (ptDNA) are circular, double-stranded polymers with essential genes for organelle function. MtDNA produces energy, whereas plastid ptDNA photosynthesizes (Oldenburg and Bendich, 2015). Maternal inheritance is the predominant pattern for mtDNA and ptDNA (Sato and Sato, 2013; Sakamoto and Takami, 2024), although biparental or paternal transmission has been observed (Weihe et al., 2009; Shen et al., 2015; Sakamoto and Takami, 2024). Both mitochondrial DNA (mtDNA) and plastid DNA (ptDNA) have higher mutation rates in comparison to nuclear DNA and can experience gene transfer events (Cui et al., 2021).

An extensive search for HGT in parasitic systems belongs to Orobanchaceae has been conducted, relying primarily on phylogenetic approaches. HGT is therefore suspected when a DNA sequence from a parasite is phylogenetically aggregated with its host instead of its nearest relatives. Examining eight host species in Fabaceae and six in Poaceae, no HGT events were detected in the facultative parasites in *Pedicularis keiskei*, *Phtheirospermum japonicum*, and *Melampyrum roseum* (Kado and Innan, 2018). In contrast, over 100 events were documented in the obligate holoparasites *Orobanche minor* and *Aeginetia indica* (Kado and Innan, 2018), indicating that the amount of HGT is directly proportional to a higher degree of host dependency. A similar pattern emerged when studying the facultative (*Triphysaria versicolor*), obligate hemiparasite (*Striga hermonthica*), and holoparasite (*Phelipanche aegyptiaca*) (Yang et al., 2016). However, with a more significant number of host species (22 representatives of land plants), the authors noted three potential events in the facultative parasite (Yang *et al.,* 2016). Furthermore, convergence is observed in the horizontally acquired sequences of two unrelated parasites: one belonging to the Convolvulaceae and the other belonging to the Orobanchaceae (Yang *et al.,* 2019). Therefore, it is likely that HGT genes, which are expressed and advantageous for the parasite, are preserved during evolution. In contrast, those that do not benefit the host are eliminated.

The high occurrence of intronic sequences and sections surpassing 100 kb, which preserve synteny between host and parasite sequences, indicates that the principal method for gaining sequences from the host includes processes mediated by DNA rather than mRNA via retro-transfer (Mower et al., 2004; Xi et al., 2012; Kado and Innan, 2018). While RNA exchange occurs through the haustorium (Westwood et al., 2009; Leblanc et al., 2012; Kim et al., 2014; Van and Alamy, 2018; Kaga et al., 2020; Zangishei et al., 2022), however, one possible explanation for the infrequent occurrence of RNA-mediated HGT is that the mRNA must undergo reverse transcription to DNA before it can be incorporated into a plant genome. However, the efficiency of this reverse transcription process is exceedingly low. Another factor is the quick turnover of RNA in comparison to DNA, making it susceptible to rapid destruction by RNases. The mechanism underlying the incorporation of host-derived DNA into the parasite genome remains unclear. The movement of large DNA fragments is not limited to parasites. This phenomenon is also found in grafted tissues of nonparasitic plants (Stegemann and Bock, 2009; Stegemann *et al.,* 2012). Indeed, the haustorial connection closely resembles graft junctions, as both structures are involved in the vascular connection between two organisms (Kokla and Melnyk, 2018; Kurotani *et al.,* 2020). Thus, close association is likely crucial in transferring DNA fragments.

HGT can be a powerful evolutionary tool for plants. It allows them to acquire new genes, potentially introducing novel functionalities that improve their overall fitness. A large-scale study revealed that green plants (Archaeplastida) obtained many of their Glycosyl Hydrolase (GH) genes through HGT, primarily from bacteria and fungi (Kfoury et al., 2024). These enzymes are crucial for breaking down carbohydrates and are essential for various cellular processes (Garron and Henrissat, 2019). Interestingly, the acquisition of these “foreign” genes was followed by a shift in how GH enzymes were distributed within plant cells (Kfoury *et al.,* 2024). There was a significant increase in GH proteins targeted to the extracellular space (Kfoury *et al.,* 2024). This strategic positioning likely played a key role in the diversification of plant cell wall polysaccharides and the development of more effective defense mechanisms against pathogens. The impact of HGT for parasitic lifestyle was documented in various organisms, including nematodes (Haegeman et al., 2011) and oomycetes (Richards et al., 2011). These gene transfers involve incorporating genes that can degrade plant cell walls. This suggests that the evolution of parasitic life in plants might have occurred via multiple acquisitions of sequences from other species, fungi being a particular focus (Richards *et al.,* 2011; Alexander et al., 2016). The nuclear gene HGT played a relevant role in some parasitic plants, while the horizontal transfer of mitochondrial genes seems even more widespread (Davis and Xi, 2015; Zervas et al., 2019). These findings underscore the substantial degree of HGT in the mitochondrial genome of parasitic species, potentially involving genes acquired from their hosts over evolutionary timescales. However, the precise biological functions of these transferred genes in the context of plant parasitism necessitate further in-depth studies.

Interestingly, it was reported that two unrelated parasite species, *P. aegyptiaca* and *C. australis*, acquired genes encoding for Strictosidine Synthase-Like (*SSL*) from Brassicaceae independently (Zhang et al., 2014). The function of these *SSL* genes remains to be revealed. Significantly, the *SSL* genes found in parasitic plants are under positive selection and exhibit continuous transcription throughout various phases of development (Zhang *et al.,* 2014). This indicates that the foreign *SSL* genes in these species may have contributed to their adaptation to a parasitic lifestyle or environment and subsequent evolution.

## Paradox of genome size of parasitic plant species

**Figure 1 -:**
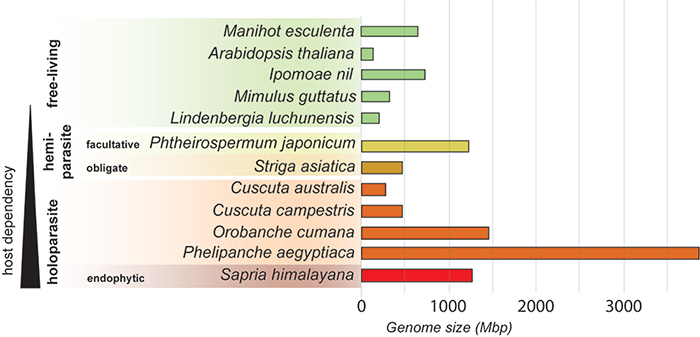
Genome size of parasitic plants compared to nonparasitic plants. Only those with fully sequenced and assembled genomes. The arrangement reflects the degree of host dependence. At the top, in green, are the free-living plants, which include *Manihot esculenta* (Alaba et al., 2014), *Arabidopsis thaliana* (Lamesch et al., 2012), *Ipomoea nil* (Hoshino et al., 2016), *Mimulus guttatus* (Hellsten et al., 2013) and Lindenbergia luchunensis (Xu et al., 2022). In light yellow is the facultative hemiparasite *Phtheirospermum japonicum* (Cui et al., 2020)- the obligate hemiparasite *Striga asiatica* (Yoshida et al., 2019) in dark yellow. Holoparasites are depicted in orange/red and include *Cuscuta australis* (Sun et al., 2018), *C. campestris* (Vogel et al., 2018), *Orobanche cumana* (Xu *et al.,* 2022), and *Phelipanche aegyptiaca* (Xu *et al.,* 2022). Lastly, the endophytic holoparasite *Sapria himalayana* (Cai et al., 2021) is shown in red, representing the most extreme parasitism.

The variation in genome size within parasitism has prompted various studies investigating its correlation with evolutionary alterations in parasitism and patterns of host usage. A shift towards parasitism is generally associated with a shrink in genome size, with parasites showing smaller genomes than nonparasitic organisms (Poulin and Randhawa, 2015; Coghlan *et al.,* 2018). However, this generalization does not hold true for all parasitic organisms; the genomes of parasitic plants serve as a counterpoint (Lyko and Wicke, 2021). Parasitic plants may exhibit larger genomes (Figure 1). For example, the highly host-dependent endophytic parasite, *Sapria himalayana*, has a genome size of 1,280 Mbp, which is on par with that of the facultative hemiparasite *P. japonicum,* which has a genome of 1,227 Mbp (Figure 1). Similarly, the holoparasites *P. aegyptiaca* and *Orobanche cumana* exhibit even larger genomes, with sizes of 3,877 Mbp and 1,463 Mbp, respectively (Figure 1). This is in stark contrast to their free-living relatives, such as *Mimulus guttatus* with a 313 Mbp genome and *Lindenbergia luchunensis* with a 212 Mbp genome (Figure 1). This inconsistency necessitates further investigation into the factors influencing genome size variation within parasitic plant lineages.

One important consideration when interpreting genome size data for parasitic plants depicted in Figure 1 lies in the inherent challenges associated with sequencing and annotating exceptionally large genomes. These technical difficulties often lead researchers to prioritize species with more manageable characteristics, such as smaller genomes, lower heterozygosity (genetic variation within an individual), reduced repetitive DNA content, and a lack of polyploidy (multiple sets of chromosomes). Supporting this notion is the observation that despite significant advancements in sequencing technology and a substantial increase in the number of sequenced plant species over the past two decades, there appears to be a relative stagnation in the range of reported genome sizes. The minimum, maximum, and median values observed in the last three years (12.40 Mb, 27.60 Gb, and 489.65 Mb, respectively) are not drastically different from those documented in the preceding twenty years (Xie et al., 2024). This lack of significant change in the spectrum of sequenced genome sizes suggests a potential bias towards species with more easily analyzed genomes (Xie *et al.,* 2024). Consequently, the data presented in Figure 1 might not fully capture the true extent of genome size variation within parasitic plants, particularly for those harboring exceptionally large genomes. 

**Figure 2 -:**
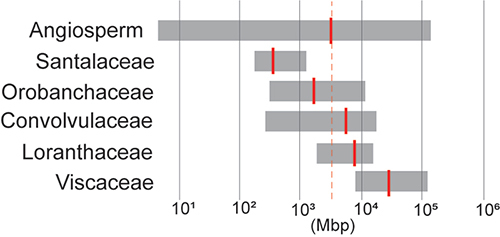
Estimated genome size (C-value) of parasitic plants. The C-values were obtained from Plant DNA C-values Database (Leitch *et al.,* 2019) maintained by Kew Royal Botanic Garden. The prime estimated c-value (in Mbp) from the database was compared with a list of parasitic plant genera (U.S. DEPARTMENT OF AGRICULTURE, 2024). Only families that have a minimum of two genera of parasitic plant species with documented C-values in the database were examined in this analysis. The grey bars represent the range of C-values observed in each group, from the lowest to the largest, while the red bars indicate the average genome size. The dashed line indicates the average genome size of all angiosperm plants recorded in the database. For Santalaceae, the genera included were *Rhoiacarpos* sp., *Santalum* sp. and *Comandra* sp. In Orobanchaceae, the listed genera were *Bartsia* sp., *Bellardia* sp., *Cistanche* sp., *Euphrasia* spp., *Melampyrum* spp., *Nothobartsia* sp., *Odontitella* sp., *Parentucellia* sp., *Pedicularis* spp., *Phelipanche* spp., *Phelypaea* spp., *Schwalbea* sp., *Orobanche* spp., *Pedicularis* spp., *Odontites* ssp. and *Rhinanthus* spp. Convolvulaceae included *Cuscuta* spp. Loranthaceae encompassed the genera *Alepis* sp., *Amylotheca* sp., *Benthamina* sp., *Decaisnina* spp., *Dendrophthoe* spp., *Diplatia* spp., *Amyema* spp., *Nuytsia* sp., *Loranthus* spp., *Lysiana* sp., *Macrosolen* sp., *Ileostylus* sp., *Muellerina* spp., *Peraxilla* spp., *Sogerianthe* sp. and *Tupeia* sp. Finally, Viscaceae comprised *Viscum* spp. and *Arceuthobium* sp.

To address this potential bias, we sought to broaden the scope of our analysis by incorporating data from the Plant DNA C-value database (http://cvalues.science.kew.org/). This resource provides genome size estimates for a wider range of plant species. Restricting our analysis to families containing at least two genera with parasitic representatives, we observed a range of genome sizes within parasitic plant lineages (Figure 2) and compared them to the average genome size in Angiosperms. While some families, such as Santalaceae (average: 569 Mb) and Orobanchaceae (average: 2747 Mb), exhibit average genome sizes below the angiosperm average. However, a different observation was indicated by Piednoël et al. (2012) that suggested that within Orobanchaceae, obligate parasitic species have larger genomes than autotrophic and hemiparasitic species (Piednoël *et al.,* 2012). These divergent findings may arise from limitations in both analyses; in our case, it’s linked to the chosen methodology and the comprehensiveness of the database. Therefore, further investigation is warranted to gain deeper insights into this inconsistency. In our approach, we observed larger genomes in *Cuscuta* spp. (average: 7,028 Mb) and Loranthaceae (average: 8,849 Mb), with Viscaceae notably possessing the highest average genome size on record (44,656 Mb) (Figure 2). Our analysis reveals a remarkable diversity in genome size among parasitic plant species, challenging the previously held notion of a universal reduction in genome size associated with parasitism (Poulin and Randhawa, 2015; Coghlan *et al.,* 2018). This underscores the paradox of parasitic plant genomes being larger and more complex than anticipated (Figures 1 and 2) (Lyko and Wicke, 2021). Importantly, it also emphasizes that the observed paradox might not be universally applicable across all parasitic plant groups (Figure 2).

**Figure 3 -:**
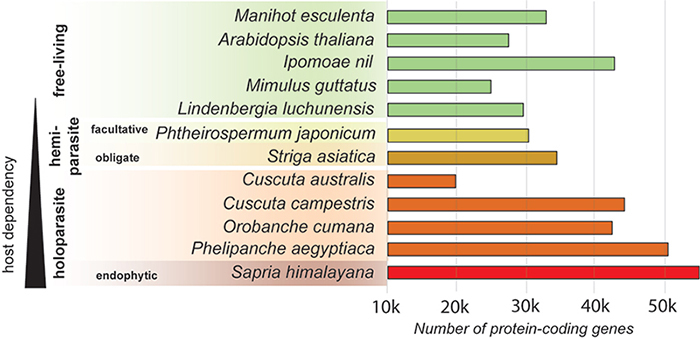
Number of protein-coding genes in the genome of parasitic plants compared to nonparasitic plants. Only those with fully sequenced and assembled genomes. The arrangement reflects the degree of host dependence. At the top, in green, are the free-living plants, which include *Manihot esculenta* (Alaba et al., 2014), *Arabidopsis thaliana* (Lamesch et al., 2012), *Ipomoea nil* (Hoshino et al., 2016), *Mimulus guttatus* (Hellsten et al., 2013) and Lindenbergia luchunensis (Xu et al., 2022). In light yellow, facultative hemiparasite *Phtheirospermum japonicum* (Cui et al., 2020). The obligate hemiparasite *Striga asiatica* (Yoshida et al., 2019) in dark yellow. Holoparasites are depicted in orange/red and include *Cuscuta australis* (Sun et al., 2018), *Cuscuta campestris* (Vogel et al., 2018), *Orobanche cumana* (Xu *et al.,* 2022), and *Phelipanche aegyptiaca* (Xu *et al.,* 2022). Lastly, the endophytic holoparasite *Sapria himalayana* (Cai et al., 2021) is shown in red, representing the most extreme parasitism.

These comparisons underscore a significant discrepancy: the size of the genome of a parasitic plant does not appear to directly correlate with its level of host dependency (Figures 2-3) which is consistent with the findings of Neumann et al. (2021). The variation in protein-coding gene numbers across different parasitic plant species (Figure 3) strengthens the evidence against a direct correlation between genome size and host dependency (Figure 3). The HGT and the mobility of large DNA fragments have been proposed as potential contributors to the expansion of genome sizes in parasitic plants (Lyko and Wicke, 2021). These mechanisms could potentially explain the observed variation between parasitic plant species. Furthermore, a recent study suggests a potential link between genome size and the type of centromere organization (Neumann *et al.,* 2021). This hypothesis is supported by observations in *Cuscuta* spp., where the transition to holocentricity (where centromeres are diffusely distributed along the chromosome) coincided with significant changes in several key features. The changes include the composition of centromeric chromatin, the makeup of repetitive DNA sequences, and even the overall number of chromosomes. The observed variation suggests that the transition to holocentricity might have been accompanied by multiple rearrangements in chromosome structure (karyotype rearrangements) (Neumann *et al.,* 2021). This finding aligns with the existing hypothesis that holocentric chromosomes exhibit a higher tolerance for chromosome fusions and fissions, potentially facilitating the observed changes in *Cuscuta*. Despite these advancements in understanding genome size variation in parasitic plants, the paradox of their larger genomes compared to typical free-living relatives remains unsolved. Research efforts should be directed towards unraveling the biological significance of these large genomes and identifying the key factors driving the observed variation within this diverse group of organisms.

## Mitochondrial genomes in parasitic plants

Plant mitochondria’s primary function is producing energy through oxidative phosphorylation (OXPHOS) (Braun, 2020). In aerobic eukaryotes, OXPHOS typically involves four respiratory-chain complexes (I to IV) and an ATP synthase (complex V). The complexes within the inner membrane of plant mitochondria facilitate electron movement through redox reactions, forming the Electron Transport Chain (ETC). This process ultimately results in a proton gradient, allowing for the synthesis of ATP through the activity of the ATP-synthase protein (Schertl and Braun, 2014). Complex I serve as the primary entry point for most electrons in the respiratory chain, facilitating their transfer from matrix NADH to ubiquinone (Braun, 2020). Complex I subunits were previously considered essential for multicellular eukaryotes’ energy production and survival. However, it has been challenged by the parasitic mistletoe, whose mitogenomes lack all the *nad* genes encoding subunits of complex I in addition to *ccmB* and *matR* (Skippingtona et al., 2015; Petersen et al., 2015; Zervas et al., 2019). Strikingly, the mitochondrial genes were not transferred to the host genome, and biochemical evidence indicates that the assembly of these respiratory complexes did not occur in the membranes of parasite mitochondria (Senkler et al., 2018). Mistletoes are not a monophyletic group (Vidal-Russell and Nickrent, 2008; Nickrent, 2011) and are presently categorized into three families in Santalales (Santalaceae, Loranthaceae, and Misodendraceae) (Nickrent, 2011; Chase et al., 2016). Currently, five species (*Viscum scurruloideum*, *V. album*, *V. minimum*, *V. crassulae*, and *Phoradendron liga*) in Santalaceae exhibit the absence of complex I (Skippingtona *et al.,* 2015; Petersen *et al.,* 2015; Zervas *et al.,* 2019), a trait not observed in *Loranthus europaeus* (Loranthaceae) (Zervas *et al.,* 2019). This suggests that the loss of complex I occurred during the evolutionary path of parasitic mistletoes within Santalaceae and not in Loranthaceae. The confirmation or refutation of this observation may be possible with further analysis of complete mitogenomes of other mistletoe species. 

The peculiar situation of mitochondria from the parasitic mistletoe plant raises questions about how OXPHOS works in these species. How can these organelles provide enough energy for the parasite? The response is that they are probably not. The organization of mitochondrial complexes in *V. album* differs from that of nonparasitic species (Senkler et al., 2018). Complexes II and IV are often coupled to form a supercomplex, whereas complexes II and V are in smaller amounts (Senkler *et al.,* 2018). This reorganization seems to impact the activity levels in the respiratory chain, resulting in reduced ATP levels in *V. album* compared to nonparasitic plants. The impaired energy production seems to be compensated by a rise in sugar intake from the host (Maclean et al., 2018). 

Aside from their essential role in energy metabolism, plant mitochondria also have a vital function in other cellular processes, including apoptosis (Scott and Logan, 2008) and cell proliferation (Kianian and Kianian, 2014). In addition, these organelles play a role in regulating signaling pathways that include crucial secondary messengers such as calcium signaling and reactive oxygen species (ROS) (Logan, 2006; Scott and Logan, 2008; Schwarzländer and Finkemeier, 2013; van Aken, 2021). The mitogenomes of parasitic plants were shown to include foreign sequences derived from several host plant species’ nuclear and mitochondrial genomes (Bellot et al., 2016; Sanchez-Puerta et al., 2019; Cusimano and Renner, 2019). The presence of HGT in the mitogenome raises concerns about its potential influence on parasitism efficiency. An initial observation attempting to address this question suggests that there is seemingly no correlation between the degree of the parasite’s reliance on the host and the frequency of HGT events in the mitogenomes. Notably, there are significant variations of host-derived sequences in mitogenomes across parasitic plants. For example, in *Rafflesia cantleyi* (Rafflesiaceae), up to 40% of the mitochondrial genes are acquired from their host’s mitochondrial genome (Xi et al., 2013).

Another example is *Ombrophytum subterraneum* (Balanophoraceae), with 14% of mitogenome acquired from the host species (Roulet et al., 2020). However, the most striking example is found in *Lophophytum mirabile* (Balonophoraceae), where 80% of the mitochondrial sequences are derived from the hosts (Sanchez-Puerta et al., 2017; Sanchez-Puerta *et al.,* 2019). A shared characteristic among them is that most foreign genes have complete reading frames and are actively transcribed. Host-derived sequences frequently replace their native homologs in parasitic organelles, appearing in clusters as large fragments while maintaining the synteny with their donor sequence. Therefore, this evidence suggests that the transfer occurs in DNA molecules, potentially through a homologous recombination process (Mower et al., 2004; 2010). 

Based on this logic, for recombination to occur, DNA fragments must be able to move between host and parasite cells. The structure of certain mitogenomes reveals a clue to the potential mechanism. In the highly HGT-prone parasite *L. mirabile*, its mitogenome (822 kb) is segmented into 54 subgenomes-circular chromosomes ranging from 7.2 to 580 kb, with 29 of them carrying intact genes (Sanchez-Puerta et al., 2017). Three other holoparasitic plants in Balanophoraceae, *Rhopalocnemis phalloides* (Yu et al., 2022), *Ombrophytum subterraneum* (Roulet et al., 2020) and *Thonningia sanguinea* (Zhou et al., 2023), are reported to consist of 21, 54 and 18 minicircular chromosomes, respectively. These small circular chromosomes share a conserved region containing a replication origin, implying they may replicate independently. The exact mechanism of this replication remains elusive. One potential explanation is the rolling circle mechanism (Yu *et al.,* 2022), a strategy previously demonstrated in yeast DNA (Prasai *et al.,* 2017), worms (Lewis et al., 2015), and other flowering plants (Backert et al., 1996; Backert, 2002). The multichromosomal mitochondrial genome is not a unique property observed in parasites. In soybeans, for example, it is estimated that the mitochondrial DNA is segmented into small circular structures (Synenki et al., 1978). The genus *Silene* (Caryophyllaceae) has been shown to contain the most extensive and most intricate multichromosomal mitogenomes, distributed into 24 chromosomes. However, many of them do not possess the ability to code for proteins. In addition, *Amborella trichopoda* has a total of five circular chromosomes (Rice et al., 2013). Three chromosomes are found in *Populus simonii* (Salicaceae) (Bi et al., 2022) and two in hybrid sugarcane (Shearman et al., 2016). However, the close connection between a parasite and its host via the haustorium may serve as a channel to transmit foreign autonomous chromosomes across species. 

The prevalence of a substantial amount of HGT described for *L. mirabile* and *R. cantleyi* is not a universal trend among parasitic plants. HGT has little or no influence on the mitogenome in several host-parasite partnerships. For instance, in *Sapria himalayana* (Rafflesiaceae), the HGT level is approximately 0.33%, while *Castilleja paramensis* (Orobanchaceae) shows 1.5%. In the case of the hemiparasite *V. scurruloideum* (Santalaceae), the HGT level is 4.7%. Additionally, there is a lack of clear evidence of HGT in mitogenomes from *Cuscuta* spp. (Convolvulaceae) (Anderson et al., 2021; Lin et al., 2022). 

## Plastid genome

The plastid genome of parasitic plants serves as a prime example of evolutionary adaptation and genomic plasticity resulting from a relaxation of evolutionary pressures (Bromham et al., 2013). The plastid genome has undergone notable modifications in parasitic plants due to their distinctive lifestyle and evolutionary adaptations. In some species, like *Cuscuta* spp., these alterations include size reduction, changes in nucleotide composition resulting in a higher AT percentage compared to nonparasitic plants, and a bias in codon usage (Chen et al., 2024).

Heterotrophic plants, such as mycoheterotrophy and parasitic plants, experience continuous deterioration of their functional plastome, often correlated with their dependence on a host. The most significant proportion of plastid inactivation genes is in endophytic holoparasites, less often lost in facultative plants. Commonly lost genes in heterotrophic organisms include genes like NAD(P)H dehydrogenase complex (*ndh* genes), plastid-encoded polymerase (PEP), and photosynthesis genes, while the most conservative sequences are those involved in translation machinery (Wicke and Naumann, 2018). The *ndh* genes encode multiple complexes that enable cyclic electron transport across thylakoid membranes. They provide extra ATP to organisms in stressful environments to repair stroma over-reduction and maintain redox system balance in the electron transfer chain (Ma et al., 2021). The lack of *ndh* genes is reported in the plastome of the holoparasite *Cuscuta* spp. (Convolvulaceae) (Braukmann et al., 2013; Banerjee and Stefanović, 2020; 2023); and Orobanchaceae species, such as *Aeginetia indica* (Chen et al., 2020), and in the facultative parasites *Pedicularis* spp. (Li et al., 2021). In contrast, *P. japonicum*, a facultative parasite in the same tribe as *Pedicularis* (Pedicularideae), has not shown any loss of plastid genes (Li *et al.,* 2021). An extreme case of plastid genome reduction can be seen in the endophytic parasitic plant *Pilostyles boyacensis* (Apodanthaceae), which retains only seven functional genes in its plastome: *accD, rpl2, rrn16, rrn23, rps3, rps12 and PbOx* (Arias-Agudelo et al., 2019). Conversely, total loss of the plastome has been documented in Rafflesiaceae species, such as *Rafflesia lagascae* (Molina et al., 2014) and *Sapria himalayana* (Cai et al., 2021). The potential loss has also been reported in *Cuscuta* subgenus *Grammica* (Banerjee and Stefanović, 2023). 

The accelerated pace of molecular evolution in the three genomes, including the mitochondrial, nuclear, and chloroplast genomes, is a defining characteristic of the shift from autotrophic to heterotrophic (Bromham et al., 2013). This increased mutation rate in parasitic plants, which may be the result of relaxed environmental pressure, is followed by a decrease in the amount of GC content that is present in the genomes of plastids (Wicke et al., 2016; Wicke and Naumann, 2018). An abundance of A+T in plant sequences is linked to a reduction of genome complexity and a higher ratio of structural organization (Müller et al., 1999; Sundararajan et al., 2016; Bowers et al., 2022), which may be the driving force behind the influence on the reduction of the size of the plastid genome in parasitic plants. The parasitic plants *Balanophora reflexa* and *B. laxiflora* (Balanophoraceae) exhibit notably high AT-rich plastomes, with AT percentages of 88.4% and 87.8%, respectively. Notably, their protein-coding genes show an even higher inclination towards AT content, approximately 91% (Su *et al.,* 2019). In an unusual genetic twist, *B. reflexa* and *B. laxiflora* have altered their genetic code, reassigning the universally recognized stop codon TAG to code for tryptophan while still using TAA as a stop codon (Su *et al.,* 2019). It is not a unique example of the variant genetic code *Mycoplasma capricolum* similarly reassigned TGA to encode tryptophan (Yamao et al., 1985). Yet, it remains a seldom observed phenomenon (Kim et al., 2023). The presence of a TA-rich genome in these cases could be attributed to constraints related to nitrogen scarcity and energy demands. Notably, the plastid proteins of both *B. reflexa* and *B. laxiflora* do not show a reduction in nitrogen-rich amino acids or energy costs (Roy Smith, 2019; Su *et al.,* 2019).

## Reshaping the parasitic cell fate during the haustorium formation

Studying parasitic plants at the molecular level remains a challenging task. It happens because the targeted parasitic species must have certain features, such as small size, ease of cultivation, and a short life cycle. Species like mistletoes, for example, have a life cycle that exceeds three years (Reid et al., 1995), making them unsuitable for use in several daily laboratory routines. Seed production is further constrained, particularly for obligatory plants, which rely on a host organism to produce seeds. Additionally, the time and substantial budget required to develop genetic information and manipulation tools present significant obstacles. Orobanchaceae encompasses a range of plant types, including free-living plants and hemi- and holoparasitic species, each displaying varying degrees of reliance on a host. *Triphysaria versicolor* and *P. japonicum* are widely recognized as essential models in the field of parasitic plant research (Tomilov et al., 2007; Yoshida *et al.,* 2020; 2019; Ishida et al., 2011; 2015; 2017 a, b; Cui et al., 2016; Yoshida *et al.,* 2016; Spallek et al., 2017; Cui *et al.,* 2020; Masumoto et al., 2021). Currently, it is available the *P. japonicum* genome (Cui *et al.,* 2020), multiple transcriptome datasets (Ichihashi et al., 2015; 2016; Cui *et al.,* 2016), genetic transformation (Ishida *et al.,* 2011), and *in vitro* assays of haustorium formation make *P. japonicum* (Ishida *et al.,* 2017a) an attractive model organism for studying the mechanisms underlying cell destiny and cellular reshaping in the development of parasitic organ haustorium. 

*Phtheirospermum japonicum*

*P. japonicum* is a facultative root hemiparasite that attaches to host plants through the development of lateral haustoria (Westwood et al., 2010). Host root compounds trigger host perception of haustorium-inducing factors (HIFs) by the *P. japonicum* root, such as 2,6-dimethoxybenzoquinone (DMBQ), lignin units, and flavonoids (Goyet et al., 2019), which are derived from the lignin biosynthetic pathway (Cui et al., 2018). After signal perception of HIFs by *P. japonicum* root, the prehaustorium begins through divisions and expansion of the epidermal and outermost cortical cells regulated by the auxin biosynthesis gene *PjYUC3* (Ishida et al., 2016). This regulation promotes the formation of haustorium hairs that differentiate from epidermal cells (Cui *et al.,* 2016), which secrete mucilage that helps the parasite attach to the host surface (Yoshida *et al.,* 2016). The cell specificity of the haustorium hairs has been demonstrated by an *AtPGP4* epidermis marker gene (Wakatake et al., 2018). In the haustorium organogenesis, the epidermis, endodermis, and most cortical cells of the parasite roots are genetically reprogrammed, originating new cell fates that cell identities confirmed by cell type-specific marker genes (Wakatake *et al.,* 2018). 

**Figure 4 -:**
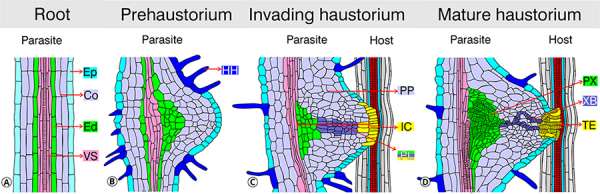
The progression of haustoria formation in *P. japonicum* (A) Initially, the figure on the left illustrates the root’s anatomical layers without a host, highlighting its independent structure. (**B**) The prehaustorium phase marks the beginning of haustorial development. (**C**) This phase transitions into the invading haustorium stage, where the haustorium starts to penetrate the host tissue. (**D**) The process culminates in the mature haustorium stage, establishing a vascular connection with the host and facilitating nutrient exchange. The diagram labels various anatomical features, including the epidermis (Ep), cortex (Co), endodermis (Ed), vascular system (VS), haustorial hairs (HH), paratracheal parenchyma (PP), procambium-like cells (PL), intrusive cells (IC), tracheary elements (TE), xylem bridge (XB), and plate xylem (PX), to illustrate the complex interactions and transformations involved in haustoria development.

In the early haustorium development stages, the epidermal cells of the haustorium apex lose their identities and differentiate into elongated intrusive cells with palisade-like shapes and thin walls, which invade the host root tissues toward the vascular system (Wakatake et al., 2018) (Figure 4). The intrusive cells express intrusive-cell-specific genes, including *INTRUSIVE CELL-SPECIFIC LEUCINERICH REPEAT RECEPTOR-LIKE KINASE 1 (ICSL1), GERMIN-LIKE PROTEIN 1 (GLP1)*, and several Subtilase encoding genes (*SBTs*) (Ogawa et al., 2021). The Subtilase activity may be important for intrusive cell formation and later haustorium development. In addition, the ethylene signaling genes *ETHYLENE INSENSITIVE 2 (EIN2)* and *ETHYLENE RESPONSE 1(ETR1)* are also involved in host invasion by regulating cell division and differentiation of the intrusive cells within the haustorial apical region (Cui et al., 2020). The invasion of the host tissues by the intrusive cells is thought to depend on the secretion activities of cell-wall-modifying enzymes such as pectin methylesterases (PME) and their inhibitors (PMEI), as well as proteases, glycosidases involved in the loosening and degradation of the cell walls (Mitsumasu et al., 2015). Currently, have been demonstrated that several *PjPME* and *PjPMEI* genes are upregulated specifically during haustorium development, which promotes the high PME activity and the low methyl esterification of homogalacturonans in the intrusive cell walls during invasion of the *Arabidopsis thaliana* host tissues (Leso et al., 2023). When the intrusive cells reach the host vasculature, some intrusive cells, the endodermis, and outmost cortical cells lose their identities, differentiating into procambium-like cells (Wakatake *et al.,* 2018) (Figure 4). The ultrastructural features have been demonstrated in two distinct groups of procambium-like cells (Figure 4). Those at the base and middle of the haustorium are highly vacuolated.

In contrast, those next to the host plant (originates of the intrusive cells) contain large cytosols with many atypical membrane structures (Figure 4). The peculiar differences also exist in terms of marker procambium gene expressions, such as *P. japonicum* orthologous, the *HOMEOBOX PROTEIN 15a (PjHB15a)*, *PjHB8*, and *WUSCHEL-RELATED HOMEOBOX 4 (PjWOX4)* are expressed widely in all regions of the procambium-like cells. Still, the expression of *PjHB15b* is restricted to the middle and basal regions (Wakatake et al., 2018). Procambium-like cells originate from the intrusive cells that differentiate into tracheary elements. The procambium-like cells on the basal regions of the haustorium near the parasite’s root xylem develop a mass of tracheary elements called plate xylem (Wakatake *et al.,* 2020) (Figure 4). Around the procambium-like cells in the middle region, the paratracheal parenchyma originated from the cortical cells of the parasite root and has cells with dense cytosol, several organelles, and thin primary cell walls (Masumoto et al., 2021). Moreover, its function is not clear yet. 

In the mature development stage, the procambium-like cells of the middle region of the haustorium differentiate into tracheal elements, establishing a xylem bridge between the parasite and the host (Yoshida *et al.,* 2016). The expression of the procambial markers is retained in actively dividing cells surrounding the xylem bridge, indicating that these cells maintain meristemic activity for tracheary element differentiation (Wakatake et al., 2018). The formation of the xylem bridge has been confirmed by marker gene expression *PjHB15a* and *CELLULOSE SYNTHASE CATALYTIC SUBUNIT 7 (PjCESA7)* that encodes a xylem-specific hemicellulose synthase for secondary cell wall synthesis (Wakatake *et al.,* 2018). In addition, the co-expression of *PjCESA7* and *DR5* suggests that auxin concentration is associated with tracheary element differentiation regulated by auxin transporters, PINs, and LAXs (Wakatake *et al.,* 2020). In this case, the expression of *PjPIN1, PjLAX1,* and *PjLAX5* directs the auxin flow from the haustorium apex toward the plate xylem formation site.

In contrast, the expression of *PjPIN9* and *PjLAX2* promotes auxin flow from the plate xylem formation site toward the haustorium apex, maintaining a high auxin gradient in the middle region (Wakatake et al., 2020). The xylem bridge, including cytokinins, is essential in transporting molecules from the host to the parasite (Spallek et al., 2017). Although the *P. japonicum* haustorium does not form the phloem connection to the host, the sieve element differentiation marker *ALTERED PHLOEM DEVELOPMENT (APL)* is expressed next to the plate xylem, suggesting that haustorium lacks characteristic phloem cells (Wakatake *et al.,* 2018).

## Impact of Environmental Factors on Plant Parasitism

In the twenty-first century, climate change emerges as one of the foremost threats to global food security, inducing challenges such as drought, desertification, salinization, flooding, and elevated temperatures. The repercussions are more pronounced in economically and socially vulnerable regions, particularly those susceptible to desertification and higher temperatures. This is notably evident in areas where parasitic weed infestations are prevalent, specifically within the genera *Cuscuta*, *Striga*, *Orobanche*, and *Phelipanche*. Consequently, parasitic plants are a significant threat to global food security. Nevertheless, there is limited knowledge regarding the impact of environmental factors on plant parasitism, raising concerns in the context of evolving global conditions and their potential effects on economically vital crops (Samejima and Sugimoto, 2018; Fernández-Aparicio et al., 2020). This predicament is exacerbated by parasitic infestations in regions characterized by poor soil fertility, recurrent droughts, and soil nutrient depletion.

Additionally, low soil fertility is thought to impede host defenses and exacerbate the damaging effects of parasitism. For instance, Kokla et al. (2022) described the influence of nutrient availability on haustorium formation in the parasitic plant *P. japonicum*, highlighting the role of nitrogen in repressing parasitism. Furthermore, the study by Miller et al. (2003) found that the infection by the parasitic mistletoe *Amyema miquelii* increased as water and salinity stress in the host plant *Eucalyptus largiflorens* decreased, while Evans and Borowicz (2013) reported that drought stress enhanced damage of the parasitic plant *Cuscuta gronovii* on its host *Verbesina alternifolia*, underscoring the diverse effects of stressors on parasitism and host tolerance. Moreover, the research by Rodenburg et al. (2023) revealed that fertilization benefits the facultative parasitic plant *Rhamphicarpa fistulosa*, while gains by the infected host *Oryza sativa* are marginalized, emphasizing the differential responses of parasitic plants and their hosts to nutrient availability. Thus, the available literature indicates that nutrient availability can significantly influence the dynamics of parasitic plant-host interactions, impacting plant growth, defense mechanisms, and the prevalence of parasitism. However, the specific effects of resource availability on the tolerance of host plants to parasitism remain an area that requires further investigation, particularly concerning the interactive effects of parasitism intensity with water and nutrient availability on the growth and defense of host plants. 

The relationship between temperature and plant parasitism highlights the intricate interplay between climate factors and the prevalence of parasitic infections in plants (Phoenix and Press, 2005; Rafferty et al., 2019). Examining how warming impacts the hemiparasite *Castilleja sulphurea* and its host, *Bouteloua gracilis,* reveals that climate change uniquely affects species interactions (Rafferty *et al.,* 2019). Warming led hosts to produce more below-ground biomass, yet the parasite’s presence curtailed this subterranean growth in the host. Moreover, the parasite experienced a more significant boost in above-ground biomass when attached to the warmed host, alongside a rise in the count of haustoria under elevated temperatures (Rafferty *et al.,* 2019). Over 13 years, David Bell and colleagues analyzed 84 locations throughout the USA, monitoring the characteristics of almost 1,400 individual trees. They discovered that warmer and drier conditions amplify mistletoe’s impact (*Arceuthobium tsugense*) on the growth of its host, *Tsuga heterophylla,* and contribute to higher mortality rates (Bell *et al.,* 2020). The research indicates that parasitic plants could amplify the adverse effects of climatic stress, heightening ecosystem vulnerability to the losses in productivity and increased mortality triggered by climate change. A key factor for this intensified impact on hosts might be that elevated temperatures influence plant physiology, compromising their defense capabilities and making them more prone to pathogen invasions (Dropkin, 1969; Wang et al., 2009; Cohen and Leach, 2020). Additionally, temperature fluctuations and changes in precipitation patterns may offer optimal conditions for spreading specific plant parasites, thus increasing the stress on plant health (Watson et al., 2022). 

## Conclusion

To understand how the climate will affect biodiversity in the future, we need to investigate more than just the direct effects of global warming. We also need to investigate the complex indirect effects of species interacting with each other. This method should include in-depth studies of ecophysiological traits like heterotrophy, autotrophic carbon gain, and how efficiently hosts and parasites use water. These are important for understanding how hosts and parasites adjust to changing climate conditions. However, understanding the molecular process behind the parasitism emergency in flowering plants is crucial to learn more about how environmental factors affect plant parasites. The large amounto of OMICS data provides us with helpful information. One example is how nucleic acid molecules move between parasite plants and their hosts. It illustrates how little we know about how stable transferred sequences are in genomes, how well they integrate into germline cells, and how this affects evolutionary patterns and genomic architecture. Many studies have been done on plastid genomes, but not many have been done on the mitochondrial genomes (mitogenomes) of plants that live on other plants. The development of long-read sequencing technologies is a bright spot because they could shed light on the complicated processes of mitogenome evolution and help us learn more about the complex biological and evolutionary processes that control the life cycles of parasitic plants. This multifaceted method of studying how climate change and plant parasitism affect each other is essential for scientific progress and for developing good conservation plans to protect our planet’s biodiversity in the face of ongoing environmental problems. It also helps answer critical biological questions about the evolution of parasitic plants and their interactions with their hosts.
